# Chronic renal impairment predicts oncological outcomes in UTUC patients undergoing RNU

**DOI:** 10.1186/s12894-024-01627-0

**Published:** 2024-11-13

**Authors:** Chris Ho-Ming Wong, Kang Liu, Hongda Zhao, Kubilay Sabuncu, Rahim Horuz, Selami Albayrak, Maria del Pilar, Laguna Pes, Jean de la Rosette, Jeremy Yuen-Chun Teoh

**Affiliations:** 1 S.H. Ho Urology Centre, Department of Surgery, Faculty of Medicine, The Chinese University of Hong Kong, Hong Kong, China; 2 Department of Urology, Medical University of Vienna, Vienna, Austria; 3 Department of Urology, Medipol Mega University Hospital, Istanbul Medipol University, Istanbul, Turkey

**Keywords:** Upper tract urothelial carcinoma, UTUC, Nephroureterectomy, Chronic renal impairment, Survival outcomes

## Abstract

**Objective:**

This study aims to explore the relationship between pre-existing renal impairment and oncological outcomes in upper tract urothelial carcinoma (UTUC) patients treated with radical nephroureterectomy (RNU) using data from a multicentre international registry.

**Patients and methods:**

Data on non-metastatic UTUC patients who underwent RNU were obtained from the Clinical Research Office of the Endourology Society Urothelial Carcinomas of the Upper Tract (CROES-UTUC) Registry. Patients were categorised into normal pre-operative renal function and chronic renal impairment (CKD) groups, with CKD defined as an estimated glomerular filtration rate below 60 mL/kg/1.73 m². Kaplan–Meier survival analysis was employed to investigate disease-free survival (DFS) and overall survival (OS). Multivariable Cox regression analysis was conducted to identify confounding variables.

**Results:**

A total of 1393 patients diagnosed with UTUC who underwent RNU between 2014 and 2019 were analysed. 875 patients (62.4%) had normal renal function, whilst 528 patients (37.6%) had CKD prior to RNU. The two groups had similar proportions of bladder cancer history, comparable cardiovascular comorbidity, similar tumour stage, and comparable proportions receiving laparoscopic or robotic-assisted RNU. In multivariable cox regression analysis, CKD was found to be associated with inferior DFS (HR = 1.419; 95%CI = 1.060–1.898; *p* = 0.019). Upon multivariable analysis, pre-existing renal impairment and higher T stage (HR = 4.613; 95%CI = 1.829–4.712; *p* < 0.001) and the use of adjuvant chemotherapy (HR = 1.858; 95%CI = 1.229–2.807; *p* = 0.003) were also found to associate with worse DFS. Significant cardiovascular disease and higher T stage were associated with worse OS. Existing renal impairment at baseline did not have any significant associated with OS.

**Conclusion:**

In this multicentre registry, preoperative chronic renal impairment was identified as an independent predictor of inferior DFS in patients undergoing RNU for non-metastatic UTUC. Preoperative CKD could serve as a clinical predictor of poorer oncological outcomes.

## Introduction

Upper tract urothelial carcinoma (UTUC) is a rare but aggressive malignancy, accounting for approximately 5–10% of all urothelial cancers [1]. The gold standard treatment for localised UTUC is radical nephroureterectomy (RNU) with bladder cuff excision [2], which confers a survival benefit in patients irrespective of age or comorbidities [3]. Despite adequate surgical resection and adjuvant chemotherapy in selected high-risk patients, the prognosis of UTUC patients is not favourable, with a 5-year recurrence rate reaching up to 30% [4]. This necessitates a deeper understanding of the prognosis of this patient group and the relevant factors that may come into play. Existing studies have demonstrated that factors including previous malignancy [5], tumour characteristics such as stage, grade, and lymph node involvement [6], and patient characteristics such as age and BMI are prognostic factors for worse oncological outcomes [7].

Recently, chronic kidney disease (CKD) has been proposed as a predictor of inferior postoperative outcomes. A correlation has been demonstrated between renal insufficiency and other urological cancers. Studies have suggested that CKD is related to an increased recurrence of bladder and prostate cancer [8, 9]. It has also been found to be associated with a higher likelihood of papillary histology in renal cell carcinoma [10]. In this context, the evidence of the impact of CKD on UTUC prognosis is weaker. In the short term, it has been related to poorer perioperative recovery with increased risks of medical complications and length of stay [11, 12].The long-term implications on survival are multifaceted. CKD could render patients unfit for adjuvant therapy [13], which is indicated in high-risk UTUC patients [14]. The additional risks of postoperative complications experienced by CKD patients, such as cardiopulmonary events and acute kidney injury, may also have sequelae in the patients’ subsequent recovery [15].

To date, there is no high-quality prospective evidence evaluating the effect of pre-existing renal impairment on the long-term outcomes of UTUC survivors receiving RNU. Current cohorts have demonstrated conflicting results, with most of them being reported by endemic regions of CKD and UTUC, such as the Asian countries of Taiwan and Japan [16, 17]. There are also clinico-pathological characteristics that differ between Caucasian and Asian UTUC patients, bringing into question the generalisability of these studies. To address this knowledge gap, we conducted this multinational retrospective analysis to investigate the implication of renal impairment on the oncological outcomes of UTUC patients receiving RNU.

## Patients and methods

Data for the current analysis were obtained from The Clinical Research Office of the Endourology Society Urothelial Carcinomas of the Upper Tract (CROES-UTUC) registry. Established in 2014, it has been one of the largest real-world prospective global databases in UTUC management, with 101 countries from 29 participating centres. The registry is registered on clinicaltrials.gov (NCT02281188) [18], and the study protocol has been published [19]. The registry follows the recommendations of the Agency for Healthcare Research and Quality for the design and use of patient registries for scientific, clinical, and health policy purposes [20].

In the current study, consecutive patients aged > 18 who were diagnosed with non-metastatic UTUC and treated with RNU were included. Cases without documentation of renal function prior to operation or those with insufficient follow-up outcomes were excluded. Patients with a history of bladder cancer were not excluded. There was no standardised protocol for the diagnostic workup, operative procedures, or follow-up protocols, which were offered according to each participating centre’s standard of care.

Patient baseline characteristics, disease details, treatment information, and follow-up data were recorded. Follow-up data, including check cystoscopy and imaging findings up to 30 months from operation, were documented and analysed. Tumour grading was performed based on the 2004 and 2016 World Health Organization classification. The reported staging was based on the evaluation of the specimen (pathological grading). Data were collected using the online Data Management System, a web-based system situated at the CROES Office.

Patients with an estimated glomerular filtration rate (eGFR) below 60 mL/min/1.73m2 were classified into the CKD group, whilst the rest were classified into the non-CKD group. This dichotomous classification was based on the Kidney Disease: Improving Global Outcomes (KDIGO) consensus [21]. eGFR was calculated using the Modification of Diet in Renal Disease (MDRD) formula [22]. The primary outcomes were overall survival and disease-free survival. Recurrence refers to disease found on the side of RNU, regional metastasis, or distant metastasis following operation. Isolated recurrence of bladder tumour after RNU or metachronous local disease of the contralateral side diagnosed after RNU was not counted towards disease recurrence.

Statistical analyses were performed using SPSS version 25.0 (IBM, New York). Differences in categorical variables between groups were assessed using Pearson’s chi-square test or Fisher’s exact test as appropriate, while continuous variables were evaluated using the Mann–Whitney U test in a non-parametric fashion. Kaplan–Meier analysis was performed for disease-free survival (DFS) and overall survival (OS). Multivariable Cox regression analyses on the primary outcomes were performed for patients with and without CKD to identify any confounding factors. Factors included in the multivariable analysis would be (1) known influencers to oncological outcomes and (2) factors that were presented as baseline between-group differences with a p-value < 0.05, and (3) significant parameters identified in the univariate analysis were fitted for multivariable analyses. A p-value of < 0.05 was considered statistically significant.

## Results

1393 patients diagnosed with UTUC fulfilled the inclusion criteria and were included in the analysis. 875 of the patients (62.4%) had normal renal function, whilst 528 (37.6%) were classified into the CKD group. The median follow up duration of the cohort is 9.2 months (interquartile range = 12.7 months). The non-CKD group had a mean preoperative eGFR of 78.6 mL/min/1.73m2, whilst the CKD group had a mean value of 43.9 mL/min/1.73m2. The median value of the two groups were 75.0 mL/min/1.73m2 (IQR = 62.5–87.5) and 46.0 mL/min/1.73m2 (IQR = 38.8–53.2) respectively. Comparing the two groups, the non-CKD group was constituted of younger patients (median age = 68.0 vs. 74.0, *p* = 0.02) and had a marginally lower Charlson comorbidity score (median value = 4 vs. 5, *p* < 0.001). Tumour characteristics were comparable. Renal pelvis tumour was the most common location (48.7% in the non-CKD group and 45.8% in the CKD group). T3 was the most commonly found local staging (accounting for 27.7% in the non-CKD group and 34.1% in the CKD group, *p* = 0.125). Grade 3 histology accounted for 49.7% in the non-CKD group and 57.4% in the CKD group. The majority of the patients were managed with laparoscopic or robot-assisted laparoscopic RNU (64.0% in the non-CKD group and 67.6% in the CKD group, *p* = 0.505). Bladder cancer was found in around 17% of the patients in either group. The details are presented in Table 1. Following RNU, the median eGFR value of the non-CKD group was 60 mL/min/1.73m2 (IQR = 49.5–70.5) and the value of the CKD group was 42.3 mL/min/1.73m2 (IQR = 32.6–51.9).

**Table 1:** Patient and disease characteristics

	Non-CKD		CKD		*P* value
	*N*	%/IQR	*N*	%/IQR	
Number of patients, %	875	62.4%	528	37.6%	
Median age, IQR	68.0	13.0	74.0	13.0	0.02
Gender, %					
Male	631	72.1%	381	72.2%	0.487
Female	244	27.9%	149	27.8%	
Median BMI (m2/kg), IQR	25.6	4.9	25.1	5.2	0.07
Preoperative eGFR (mL/min/1.73 m2)					< 0.001
Mean, SD	78.6	16.9	43.9	12	
Median, IQR	75.0	25.0	46.0	14.5	
Median Charlson comorbidity score, IQR	4	2–6	5	3–8	< 0.001
Significant cardiovascular disease, %	101	11.5%	95	18.0%	0.06
History of smoking, %	533	60.9%	289	54.7%	0.02
ASA, %					
1	129	14.7%	49	9.3%	< 0.001
2	469	53.6%	232	43.9%	
3+	262	29.9%	243	46.0%	
Missing	15	1.7%	10	1.9%	
History of bladder cancer. %	143	16.3%	90	17.0%	0.767
Surgical approach, %					
Open	315	36.0%	171	32.4%	0.505
Laparoscopic or robotic	560	64.0%	357	67.6%	
Tumour laterality, %					
Left	428	48.9%	258	48.9%	0.496
Right	447	51.1%	270	51.1%	
Tumour location,%					
Ureter only	211	24.1%	140	26.5%	0.447
Renal pelvis only	426	48.7%	242	45.8%	
Multifocal	94	10.7%	51	9.7%	
Missing	144	16.5%	95	18.0%	
Tumour grading, %					
Grade 1	116	13.3%	38	7.2%	< 0.001
Grade 2	215	24.6%	118	22.3%	
Grade 3	435	49.7%	303	57.4%	
Missing	109	12.5%	69	13.1%	
pT stage, %					
Ta/is	173	20.9%	86	16.3%	0.125
T1	178	20.3%	96	18.2%	
T2	169	19.3%	95	18.0%	
T3	242	27.7%	180	34.1%	
T4	36	4.1%	27	5.1%	
Missing	77	8.8%	44	8.3%	

CKD = chronic kidney disease; IQR = interquartile range; BMI = body mass index, ASA = American Society of Anesthesiologist classification; pT stage = pathology tumour staging

**Fig. 1:**
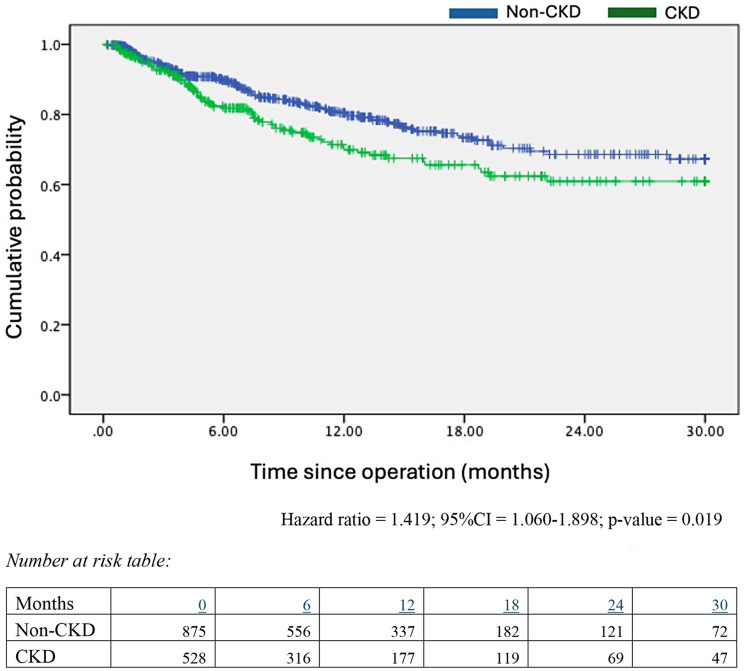
Kaplan Meier Survival Curves on disease-free survival of CKD and non-CKD cohorts

In the Kaplan-Meier survival analysis, the non-CKD group was associated with superior 30-month disease-free survival (Hazard ratio = 1.419; 95%CI = 1.060–1.898; *p* = 0.019) (Fig. 1). In the univariate analysis, pre-existing renal impairment (HR = 1.419, *p* = 0.019), history of smoking (HR = 1.373, *p* = 0.033), advanced tumour stage (HR = 2.121, *p* = 0.002), grade 3 tumour histology (HR = 2.340, *p* = 0.003), and use of adjuvant chemotherapy (HR = 2.274, *p* < 0.001) were found to be statistically significant predictors of inferior outcomes. In the multivariable analysis, existing renal impairment, tumour stage, and adjuvant chemotherapy remained statistically significant after adjustment (*p* = 0.048, 0.027, and 0.003, respectively) (Table 2).

**Table 2:** Cox regression analysis on factors associated with disease free survival

Univariate analysis	Effect size	95% CI		*P* value
Existing renal impairment	1.419	1.06	1.898	0.019
Smoking history	1.373	1.026	1.837	0.033
Age > 70	1.36	0.993	1.862	0.055
Female	1.127	0.813	1.564	0.473
Charlson comorbidity score	1.091	0.998	1.193	0.055
Significant cardiovascular disease	1.174	0.804	1.716	0.406
pT stage (Ta/is/1 as reference)				
T2	2.121	1.331	3.379	0.002
T3	3.758	2.563	5.509	< 0.001
T4	7.8	4.33	14.053	< 0.001
Tumour grade (Grade 1 as reference)				
Grade 2	0.754	0.382	1.49	0.417
Grade 3	2.34	1.324	4.136	0.003
Multifocal disease	1.24	0.935	1.646	0.136
Variant histology	0.677	0.246	1.864	0.45
Adjuvant chemotherapy	2.274	1.71	3.024	< 0.001
Adjuvant bladder instillation	1.015	0.73	1.411	0.932
Previous bladder cancer	0.794	0.502	1.255	0.323
**Multivariable analysis**	**Effect size**	**95% CI**		***P*** **value**
Existing renal impairment	1.375	1.004	1.884	0.048
Smoking history	1.333	0.964	1.844	0.083
pT stage (Ta/is/1 as reference)				
T2	1.788	1.067	2.996	0.027
T3	2.936	1.829	4.712	< 0.001
T4	4.715	2.336	9.519	< 0.001
Tumour grading (Grade 1 as reference)				
Grade 2	0.538	0.266	1.09	0.085
Grade 3	1.047	0.543	2.019	0.891
Adjuvant chemotherapy	1.858	1.229	2.807	0.003

CI = confidence interval; pT stage = pathology tumour staging

**Fig. 2:**
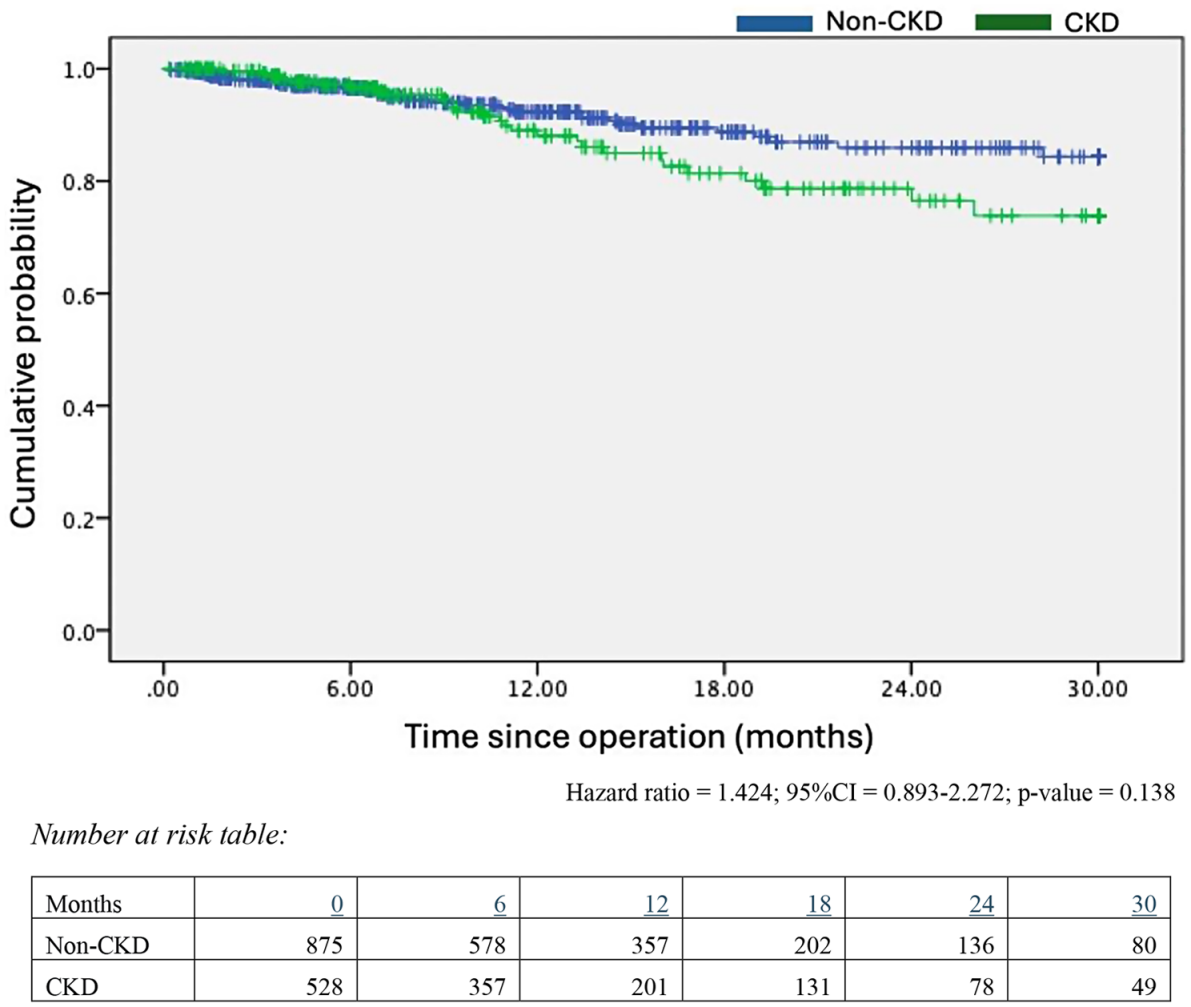
Kaplan Meier Survival Curves on overall survival of CKD and non-CKD cohorts. Hazard ratio = 1.424; 95%CI = 0.893–2.272; *p*-value = 0.138

In the analysis for overall survival, there was no association found between CKD and OS after adjustment in the multivariable analysis model (Fig. 2). A history of significant cardiovascular disease (HR = 1.892; 95%CI = 1.971–3.340; *p* = 0.028) and tumour staging (HR = 4.631; 95%CI = 1.924–11.065; *p* = 0.007) were found to be statistically significant predictors of overall survival in UTUC patients post-RNU (Table 3).

**Table 3:** Cox regression analysis on factors associated with overall survival

Univariate analysis	Effect size	95% CI		*P* value
Existing renal impairment	1.424	0.893	2.272	0.138
Smoking history	0.933	0.583	1.494	0.773
Age > 70	2.056	1.254	3.372	0.004
Female	1.214	0.712	2.071	0.476
Charlson comorbidity score	1.107	0.965	1.271	0.147
Significant cardiovascular disease	1.917	1.13	3.254	0.016
pT stage (Ta/is/1 as reference)				
T2	3.16	1.433	6.966	0.004
T3	6.028	3.086	11.772	< 0.001
T4	13.675	5.038	37.118	< 0.001
Tumour grade (Grade 1 as reference)				
Grade 2	0.543	0.172	1.712	0.297
Grade 3	2.587	1.035	6.465	0.042
Multifocal disease	1.016	0.384	2.686	0.974
Variant histology	1.177	0.163	8.509	0.872
Adjuvant chemotherapy	3.925	2.183	7.058	< 0.01
Adjuvant bladder instillation	0.927	0.475	1.81	0.825
Previous bladder cancer	0.71	0.353	1.427	0.337
**Multivariable analysis**	**Effect size**	**95% CI**		***P*** **value**
Age > 70	1.769	0.952	3.288	0.071
Significant cardiovascular disease	1.892	1.071	3.34	0.028
pT stage (Ta/is/1 as reference)				
T2	2.357	0.923	6.021	0.073
T3	4.613	1.924	11.065	0.001
T4	6.478	1.669	25.144	0.007
Tumour grading (Grade 1 as reference)				
Grade 2	0.306	0.088	1.065	0.063
Grade 3	0.763	0.252	2.308	0.631
Adjuvant chemotherapy	1.858	0.762	4.535	0.173

CI = confidence interval; pT stage = pathology tumour staging

## Discussion

From our study, it is found that CKD is an independent predictor of disease-free survival after adjusting for potential confounders. This echoed with existing cohorts [23–25]. Our findings further corroborated this postulation, with data yielded from a real-world database, contributed by centres from different level of expertise. It could be possibly explained by that fact that the event rate that accounts for OS maybe too small to be detected in the current follow-up duration. This is especially true in low risk UTUC patients, who may become long survivors following RNU. Shall the data mature with a longer follow-up, the effect of CKD could become statistically significant at a later time point. In summary, CKD is associated with a worse prognosis in RNU patients. Overall, the finding is obviously of clinical significance since CKD appears to be associated with a worse prognosis in RNU patients.

There may be several reasons for a worse DFS following RNU in CKD patients. First, Renal impairment may be a cause for possible treatment restriction following RNU and this was postulated to contribute to worsened outcomes. The POUT trial was a landmark trial suggesting that the adoption of Gemcitabine-Cisplatin or Gemcitabine-Carboplatin chemotherapy for high-risk UTUC patients brings about a 50% relative risk reduction in disease recurrence or death [26]. However, in the real-world setting, physicians are often faced with the dilemma of RNU-induced CKD. Lane and colleagues analysed 336 UTUC patients and noted that only 48% of patients were eligible for cisplatin-based therapy. Following RNU, the number fell to 22% [27]. A considerable number of patients who were eligible for chemotherapy would no longer be fit after surgery.

On the other hand, there was less convincing evidence to support giving neoadjuvant chemotherapy (NAC) rather than adjuvant therapy. Prospective phase II trials have demonstrated variable complete pathological response rates in patients receiving NAC followed with RNU [28, 29]. In a 2019 systematic review of 4 retrospective studies involving 318 patients, Kim et al. concluded the benefit of NAC in terms of overall, cancer specific and progression free survival [30]. Another systematic review and meta-analysis by Leow et al. in [31] also concluded similar findings. However, we are still lacking well designed level 1 RCT evidences for NAC and thus the suggestion for NAC usage remained level 2 at best. Also, the challenge of acute local staging of UTUC abounds, when considering the indication of NAC. Without specimen review from RNU, it would not be feasible to confidently comment on the disease staging purely from preoperative imaging. Offering NAC to this group of patients comes with a risk of overtreatment. A feasible way out would be to maximise the adoption of nephron-sparing surgery in order to maximally preserve renal function, amidst an era of technological advancements with better flexible ureteroscopes, more efficient laser options, and wider penetration of robotic surgery platforms.

Secondly, explanations at the molecular level as to how chronic renal impairment is related to worse prognosis of UTUC were also suggested. Frequent exposure to renotoxic agents in CKD patients may lead to pan-urothelial cancerisation [32]. CKD is also related to a systemic state of increased inflammation [33]. Activation of proinflammatory cytokines may result in impaired DNA repair and hence carcinogenesis. Uremia, which is related to severe CKD, resulted in impairment of the immune system via T cell activation and thus tumour progression. Whilst these explanations are less modifiable and of lesser interest to surgeons, they highlight the multifactorial mechanism in the relationship between CKD and UTUC carcinogenesis.

The proportion of CKD patients was significant in the current study, with 37.6% of patients identified as having renal insufficiency prior to operation. In a meta-analysis of 16 studies incorporating 3765 patients, which aimed to look at oncological outcomes, the pooled incidence of CKD was 35.9% (ranging from 11.9 to 75.3%) [23]. The current study was in line with existing data. The significant proportion of renal insufficiency patients within this patient population does raise a concern for long-term management in these patients. UTUC patients were faced with a host of risk factors for renal impairment. Urinary tract obstruction, age, and comorbidities were some of the major factors [34]. The high prevalence of renal impairment in UTUC patients should be a concern for physicians in the course of management.

The incidence of multifocal tumours was around 10% in both the CKD and non-CKD groups. The proportions of isolated ureteric tumours were around one-fourth in both groups. This pattern of disease location is different from a recent 1172-patient Taiwanese RNU cohort reported by Lee and colleagues. In their study, they found that multifocal tumours were present in more than one-third of the patients in the CKD group [16]. They also noted that multifocality alongside CKD were predictors of inferior DFS. In our study, the rate of multifocal tumours was lower, and it was not found to be a predictor of inferior prognosis. This highlighted the different clinico-pathological characteristics of UTUC manifested at the clinical level. In the Asian population, traditional herbal medicine is a common remedy. Aristolochic acid (AA) is a common ingredient found, which was associated with UTUC patients being developed in younger patients, together with more advanced CKD [35]. Whilst the current study showed a different clinical pattern compared to the Asian studies, CKD was also found to be associated with a worse prognosis. This highlighted the value of the current study, in how the primary results can be extrapolated to the wider community worldwide.

The major limitation of the current study is given by its observational retrospective nature. Hence, despite the effort in adjusting for confounders, it is not possible to identify and balance all the possible confounding factors that potentially affect survival outcomes. As the primary endpoints are medium-to-long term survival outcomes, there would unavoidably be dropout cases. The multicentre nature of the study, which includes a vast collection of cases managed with different approaches, means that a standardised follow-up protocol to unify follow-up data is not possible. The current analysis was based on a dichotomous classification of CKD. While it could facilitate easy interpretation by physicians, the investigation into the influence of difference stages of CKD was limited. While the current database intended to include a multitude of centres including both academic centres and general units, the level of expertise within participating centres varied, therefore the analysis included RNU from both high and low volume centres. Alternative propensity score may be considered but would not be ideal either, as it may render the sample size insufficient to detect statistically significant factors.

This study enharbours a major strength. To date, there is no prospective literature investigating the implications of preoperative renal impairment on the long-term survival outcomes of UTUC patients post-RNU. This is by far the largest cohort reporting on the effect of CKD on UTUC survival.

## Conclusion

Pre-existing renal impairment of eGFR < 60mL/min/1.73m2 is a predictor of worse prognosis in UTUC patients following RNU. With data from multiple institutions of verifying level of expertise and different regions, we depicted the implication of CKD on UTUC management in a real-world scenario. Physicians shall be aware that preoperative CKD could serve as a clinical predictor of poorer oncological outcomes.

## Data Availability

The datasets used and/or analysed during the current study available from the corresponding author on reasonable request.

## Abbreviations

UTUC: Upper tract urothelial carcinoma
RNU: Radical nephroureterectomy
CKD: Chronic kidney disease
eGFR: Estimated glomerular filtration rate
KDIGO: Kidney Disease: Improving Global Outcomes
MDRD: Modification of Diet in Renal Disease
CROES: Clinical Research Office of the Endourology Society
OS: Overall survival
DFS: Disease free survival
BMI: Body mass index
